# Telehealth-Enabled Emergency Medical Services Program Reduces Ambulance Transport to Urban Emergency Departments

**DOI:** 10.5811/westjem.2016.8.30660

**Published:** 2016-09-06

**Authors:** James R. Langabeer, Michael Gonzalez, Diaa Alqusairi, Tiffany Champagne-Langabeer, Adria Jackson, Jennifer Mikhail, David Persse

**Affiliations:** * The University of Texas Health Science Center, Departments of Emergency Medicine and Biomedical Informatics, Houston, Texas; † Baylor College of Medicine, Department of Emergency Medicine, Houston, Texas; ‡ Houston Fire Department, Emergency Medical Services, Houston, Texas; § The University of Texas Health Science Center, School of Biomedical Informatics, Houston, Texas; ¶ City of Houston Health and Human Services, Division Manager, Houston, Texas; || The University of Texas Health Science Center, Research Manager, Houston, Texas

## Abstract

**Introduction:**

Emergency medical services (EMS) agencies transport a significant majority of patients with low acuity and non-emergent conditions to local emergency departments (ED), affecting the entire emergency care system’s capacity and performance. Opportunities exist for alternative models that integrate technology, telehealth, and more appropriately aligned patient navigation. While a limited number of programs have evolved recently, no empirical evidence exists for their efficacy. This research describes the development and comparative effectiveness of one large urban program.

**Methods:**

The Houston Fire Department initiated the Emergency Telehealth and Navigation (ETHAN) program in 2014. ETHAN combines telehealth, social services, and alternative transportation to navigate primary care-related patients away from the ED where possible. Using a case-control study design, we describe the program and compare differences in effectiveness measures relative to the control group.

**Results:**

During the first 12 months, 5,570 patients participated in the telehealth-enabled program, which were compared against the same size control group. We found a 56% absolute reduction in ambulance transports to the ED with the intervention compared to the control group (18% vs. 74%, *P*<.001). EMS productivity (median time from EMS notification to unit back in service) was 44 minutes faster for the ETHAN group (39 vs. 83 minutes, median). There were no statistically significant differences in mortality or patient satisfaction.

**Conclusion:**

We found that mobile technology-driven delivery models are effective at reducing unnecessary ED ambulance transports and increasing EMS unit productivity. This provides support for broader EMS mobile integrated health programs in other regions.

## INTRODUCTION

### Background

Emergency medical services (EMS) plays a vital role in the appropriate prehospital management of the nearly 250 million 911 callers each year.^1^ Both emergency departments (ED) and EMS agencies are increasingly resource-constrained, threatened by the increasing number of ambulance transports often associated with non-urgent complaints.^2^ Most EMS protocols require the transport of all 911 patients to the ED and lack incentive to transport patients to possibly more appropriate settings. As a result, resource costs are high through unnecessary transport and ED care for non-urgent primary care patients. A nationwide study estimated that the proportion of medically unnecessary EMS transports has increased 31% from 1997 to 2007 (from 13% to 17%), supporting the need for alternative models of EMS prehospital care.^3^

The American College of Emergency Physicians concludes that ambulance non-transport as well as transportation to alternate destinations may be appropriate for non-urgent patients.^4^ The same report contends that EMS systems choosing to implement such options “should develop a formal program to address these alternatives” and should occur only under physician oversight, combined with adequate education of EMS providers and a strong quality management system. Approximately 7% of EMS agencies serving the 200 largest cities in the U.S. have implemented policies allowing EMS-initiated non-transport of patients.^5^ However, there is a limited amount of research determining the safety and effectiveness of these programs.^6^

Programs that combine non-traditional techniques and technologies to redeploy units and more appropriately align patients to alternative destinations are conceptually termed “mobile integrated health” (MIH) or “community paramedicine” (CP). The difference in the models is the deployment of personnel and technology. Mobile integrated health involves technology utilization, and is defined as “the provision of healthcare using patient-centered mobile resources in the out-of-hospital environment”.^7^ Community paramedicine describes the expansion of EMS personnel roles and responsibilities more broadly in public health and healthcare delivery.^8^ Collectively, these are alternatives to traditional EMS treat-and-transport models. Alternative models tend to emphasize technology, non-ambulance-based transportation, and broader paramedic roles and responsibilities to “reduce total cost of care, provide more patient-centered care, and reduce the burden on EDs”.^9^ Most patient-centered alternative models include technology to support telehealth. Telehealth has typically been performed in rural areas or for specialized diagnoses, providing care remotely to patients that otherwise would not receive any. Formally, telehealth is the use of electronic communication to facilitate patient care between a patient and a provider working at a distance. 10–11

### Significance

Non-urgent, primary care-related incidents severely hamper the current emergency medical care system. The potential benefits of an alternative mobile integrated health program include enhancement of resource utilization, reduction of unnecessary ED visits that contribute to crowding and access to care. 12 Schaefer et al. reported a 7% reduction in ED use and 3.5% increase in community clinic use in the post-phase implementation of an alternate destination program for selected non-urgent patients.^13^ In a similar evaluation of an alternate destination program in the United Kingdom, Snooks et al. reported reduced waiting times, increased patient satisfaction, enhanced resource utilization, and shortened cycle times for ambulance services.^14^ Other studies have shown the safety of alternate methods of transport (e.g., taxi) and effectiveness of physician-directed destination programs to reduce crowding.^15^–^16^

Although there are a few documented studies of EMS alternative programs and telemedicine pilots, these are often in rural settings or in small demonstration projects.^17^ Other emergency researchers have pointed to a significant need for more comparative effectiveness studies of large-scale MIH programs.^18^

### Study Objective

The objective of this research is to compare the effectiveness of an alternative EMS telehealth delivery model relative to traditional EMS care in a large urban, American city.

## METHODS

### Study Design

We developed an observational case-control study between two groups of patients who placed emergency medical calls to 911. The intervention group (ETHAN patients) incorporated telehealth with community paramedicine, and dispositioned patients to the most appropriate level of care (e.g., hospital ED, local safety net clinic with prepaid taxi voucher, or referrals to primary care). The control group was comprised of traditional EMS patients treated and transported to local EDs per standard protocol. We measured the effect differences across a number of different measures.

### Study Setting

With a population of more than 2.2 million, the City of Houston covers an area of over 600 square miles in Southeast Texas. The city’s emergency medical services (EMS) is a division of the Houston Fire Department. Houston EMS receives over 250,000 emergency calls every year. As a fire-based EMS department, a two-person unit will respond to all EMS calls in one of the 63 ambulances, 89 engines, 39 ladder trucks, or 35 medic response vehicles located at 93 fire stations across the region. EMS services benefit all of the city’s residents, and frequently support those most in need, such as low-income mothers and children, the elderly, and Medicaid and minority populations. The program serves the region’s primary EMS population, which is comprised of approximately 30% Medicaid enrollees and 20% indigent patients.

This demand for emergency services has steadily risen over the past decade and continues to increase. Recognizing the rising costs of treating patients with non-emergent conditions, the City of Houston Department of Health and Human Services, received funding from the 1115 Medicaid Waiver pool to develop an intervention program (ETHAN), aiming to reduce the number of potentially unnecessary ambulance transports and ED visits. Initial investment of $500,000 was used for capital equipment, including the telehealth and tablet hardware and software. Approximately $1,000,000 per year for five years will also be used to cover all operational expenses of the program. The proposal was to incorporate telecommunications technologies to triage patients with non-life-threatening, mild or moderate illnesses via telemedicine with an emergency physician at the Houston Emergency Center. The EMT/paramedic on the scene would be responsible for making the determination of whether or not the situation warranted a triage intervention. If not, and the patient met inclusion criteria listed below, they would be eligible to be enrolled into the program. The paramedic would then activate ETHAN through an online call button on the tablet, which contacts the emergency physician in the base station immediately for a consultation. If the treating physician determines that the patient did not need immediate medical attention, the patient receives a referral for an appointment and follow-up care at a participating clinic the same or following day.

### Sample Determination and Participant Selection

Sample size was calculated assuming 80% power and significance level 0.05, for continuous data. We chose reduction in ambulance transportation as our primary effect, and aimed to detect a difference of 0.10 between ambulance transports for our intervention participants, assuming that the base rate of transport was 78%. We calculated a necessary sample size of approximately 2,000 total patients in both the case and control groups.

Each patient who received the intervention was matched retrospectively with a similar patient identified in the patient care record (PCR) system as a control. The patients were matched during the same period, based on individual factors, including similar primary care chief complaints, age, and gender. We matched 100% of the cases with controls, to have the identical size samples in each group. This study design allowed us to compare outcomes (e.g., % ambulance transport, as well as other clinical, economics, patient satisfaction) relative to a similar set of traditional EMS patients.

Patients selected for the program had to meet inclusion criteria, as determined by the field paramedics at time of triage. Inclusion criteria for this study were patients with full mental capacity presenting with chief complaints that were primary-care related. The most common complaint categories system were “abdominal pain,” “sick,” “injury/wound,” and “other pain.” Patients had to consent to speaking to a physician, have no obvious emergency present and vital signs within reasonable limits, and they had to be ambulatory and mobile. Inclusion criteria included the following:

- Full history and physical exam, no emergency
- Ages > 3 months
- Ability to communicate and to speak English
- Vital signs are age appropriate and within normal limits
- Chronically ill patients or persons over age 65 years may not have a fever
- Ability to care for self
- Transported in a passenger vehicle
- Pediatric patients must have access to a pediatrician.

We excluded patients if there were any urgent issues such as chest pain, acute neurological changes, or altered mental status. Other exclusion criteria included the following:

- Ongoing difficulty breathing
- Chest pain or discomfort
- Any acute neurological change
- Syncopal episode in the past 24 hours
- Temperature of >100.3 if chronically ill or 65+
- Non-trivial traumatic injury in a patient <18
- Any pediatric patient when non-accidental injury or neglect is suspected
- Any pediatric patient <18 years who has no legal guardian on site
- Any patient who refuses to participate

### Intervention Protocol

The intervention consists of the following three components: 1) telehealth capabilities between the paramedic, patient, and an EMS physician; 2) patient navigation and scheduling to contracted safety net clinics, if possible; and 3) taxi transportation and social service follow-up post incident. The intervention initiates when the first responding apparatus arrives at the incident scene, and the crew assesses the patient to make an initial determination as to the emergent status of the patient’s condition. The figure shows the study protocol flowchart.

All EMS units carried tablets to connect the patient with an emergency physician via HIPAA-compliant and secure video teleconferencing software. Telehealth services involved synchronous communication with the patient through video conferencing on the tablet. The emergency physician was able to access the patient’s medical record created at the scene, including patient’s demographics, vital signs, medical history, allergies, medications, and chief complaint. Although the community health information exchange system was available, the lack of available data for most patients prevented it from being used to access the previous hospital records of patients. The physician consulted with the patient through the tablet, and made a determination of preliminary diagnoses and treatment options.

The EMS physicians were board-certified emergency physicians who practice at local hospitals EDs and contracted for part-time shifts at the Houston Emergency Center specifically for telehealth calls. There are approximately 16 physicians employed, all with at least five years of experience and practice in one of the local hospitals. All except for the program director (who was also an MD) were contracted part-time employees working at least one shift, and the hourly compensation was between $160–$200. There was one physician on duty at all times from 8 am to 9 pm, five days per week, and 10 am to 6 pm during the weekends. Physicians were given a desk with both a computer enabled with camera and access to multiple software solutions, including the EMS patient care record (PCR) system, a clinic scheduling system, taxi activation links, and the health information exchange. All physicians were municipal employees under the City of Houston, and were covered for liability and malpractice under the city’s sovereign immunity law.

Training for the telehealth and navigation program lasted four hours, where the physicians were given technical training and instructions on the goals and objectives of the study. During the training period, the physicians test all technology components, observe multiple calls in progress, and then take calls under the supervision of a more experienced physician. Following this training, they were independent going forward, although weekly feedback and outcomes were shared by the program director.

While the video encounter was taking place, the field crew remained on scene to assist the physician with any additional information needed, such as taking a new set of vital signs or palpating the patient’s pain site. The physician, in consultation with the patient, made the final determination regarding patient disposition. Patient’s preference and input often led to the disposition to an ED rather than a clinic (although in a taxi versus ambulance). We saw no differences in patient diagnosis for those dispositioned to the ED versus a clinic.

The median number of minutes for a telehealth call was eight minutes, but ranged from 2–40 minutes (interquartile range). Since the ability to speak English was an inclusion criterion, all telehealth calls were in English as well.

### Outcome Measures

The objective of this study was to explore the relative effectiveness of a large MIH program focused on primary care-related patients, relative to traditional EMS. The primary outcome measure was utilization, measured as the proportion of ambulance transports to the ED. Ambulance utilization is considered important as it impacts local hospital EDs’ crowding, wait times, and access.

Another primary outcome metric was unit productivity, as that ultimately influences total cost of care. This was calculated as the total “back in service” time, measured by the difference in minutes between when the unit was dispatched and the unit became available to respond to a subsequent incident. Generally, the quicker the unit is available and put back in service, the more productive the crew and the ambulance. Utilization is greater if units terminate the call after initial review and observation, rather than disposition to an ED, which often requires long transport and transition times. While cost was not directly studied here, an ongoing health economics study is estimating the program’s total cost of care. Secondary measures we chose to include were quality of care (measured by mortality rates), and the experience of care (measured as post-incident patient satisfaction).

### Primary Data Analysis

We extracted all patient demographics, interventions, treatment times, dispositions, and outcomes data from the PCR system used by Houston Fire Department. We obtained all patient data in the program from January 1, 2015, through December 31, 2015, and de-identified the data after abstraction. Data were validated in a database using scripts to ensure completeness of data for all cases. We used both operational and information systems personnel at Houston Fire Department to ensure that all extracted data for both cases and controls were accurate and complete prior to inclusion in the dataset for analyses. We used descriptive analyses to determine frequencies and central tendencies. Continuous outcomes, unless otherwise stated, were compared between treatment groups with t tests. Time data were highly skewed and therefore the nonparametric Mann Whitney U test assessed median differences. We used SPSS to perform data analysis (SPSS Statistics, version 23, Armonk, NY: IBM Corp.).

This comparative effectiveness study was reviewed and approved by the institutional review board at the University of Texas Health Science Center at Houston.

## RESULTS

During the study period, 5,570 patients participated in the intervention program. There were 288,000 total EMS calls during that period. Table 1 shows the descriptive characteristics of the patients in the intervention and the matched control group.

We found a statistically significant change in alternative transport options, with a 56% absolute decrease in transport to the ED (74% for control group vs. 18% for intervention; *P*<.001). In the control group, the 26% (which did not go to the ED) ended up as non-transports. Of the non-ambulance transports, most intervention patients (n=3,293, 72% of non-transports) were offered a pre-paid taxi ride to go to a local hospital ED independently. Approximately 83% of these actually used the taxi and presented to the ED (2,733). This disposition was appropriate where patients might need care not offered by a clinic, but were not emergent enough to require immediate ED care.

There were 458 patients (8%) scheduled into one of the geographically proximate safety net clinics, usually within the day or next business day. The EMS physician was successful in securing appointments for 100% of these patients, although only 55% of them actually presented to the clinics (i.e., 45% no-show rate). There was patient follow up by telephone within a week to inquire about their appointment, and most reported their symptoms subsided as reason for missing appointment. Based on the diagnosis, we had no reason to believe that mortality was a cause for patient no-show. Fourteen patients made a follow-up call after referral to the primary care clinic for an incident within a two-day time period (<.2%), resulting in a subsequent EMS response. The remainder were referred to the patient’s own primary care physician or home care, refused care, or were provided home care instruction only. Approximately 7% (259 patients) declined to speak to an EMS physician by telehealth in the intervention group, or refused referrals to clinics, or technical or other issues prevented one of the other dispositions. Of these, technical issues represented only around 50 calls, which was primarily due to lack of wireless cellular signal in certain regions of the city. Table 2 presents the disposition rates for the intervention.

Patient satisfaction was recorded by follow-up telephone services from the City of Houston Health and Human Services caseworkers for both ETHAN and non-ETHAN patients. We attempted to contact 100% of the intervention patients by telephone, but we received approximately 10% completed survey response rate, primarily due to inactive or erroneous telephone contact information. We sampled 10% of the control group to ensure the same sample size. There was no difference in “overall satisfaction with care delivered by EMS,” with ETHAN patients reporting an 88% overall patient satisfaction rating for the EMS response, compared to 87% for the non-intervention group (p=.25). There were 10 survey questions, but the satisfaction rating used here was based on the response to the question “Overall, on a scale of 1 – 100 (where 100 is the best), how would you rate your level of EMS care?”

Since these were primary care-related incidents, there were zero mortalities reported in either of the groups during the prehospital phase for either the intervention or control groups, and consequently there was no significant differences in that measure between groups.

Most significant were the differences in EMS productivity. The median response time (from EMS notification from 911 to unit back in service time) was 39 minutes for ETHAN patients, and the median response for the control group was 83 minutes. This 44-minute reduction in medians between the groups is statistically significant (Mann Whitney *P*<.001). This equates to approximately 2.1 times greater utilization (dispatches per day) for the EMS unit than the standard EMS control group, resulting in significantly lower cost of care. Table 3 summarizes the outcome results.

## LIMITATIONS

There are several limitations to this study. An important one is the lack of randomization. Given the nature of the study and the practicality of EMS response, we used a case-control observational design. There are obvious inherent limitations in the selection of the control group, although we made every effort to match the patients based on age, gender, approximate dates, and chief complaint In addition, this study uses data extracted from multiple components of a PCR system. As with all patient record systems, the accuracy and quality of the data entered by field crews may be inaccurate or incomplete. We incorporated multiple special precautions for ensuring data quality and validity of the dataset to mitigate this limitation, including oversight from both operational and information technology personnel at the fire department.

Another limitation is that this study represents only a small subset of total EMS calls in this large city (roughly 1.9% of all calls in 2015). Since it was designed as a pilot study to assess feasibility and relative effectiveness on measures of ambulance utilization and EMS productivity, future period will use greater sample sizes. Lack of comprehensive data on post-EMS response outcomes is also a limitation. Although we found no reported deaths, we were not able to do a comprehensive search of all patients that might have died after the EMS response. We were not able to determine the effect of the ETHAN program on ED crowding across more than 60 hospitals with 1.4 million ED visits. Finally, there were few technical limitations of this telehealth system, although a very small subset of calls were aborted due to poor wireless cellular signals required to use the paramedics’ tablets in patients’ homes. As wireless networks continue to improve in the region, this should be less of an ongoing problem over time.

## DISCUSSION

To our knowledge, this study represents one of the largest, urban efforts at integrating mobile technologies and alternative patient navigation to improve EMS utilization and outcomes. As suggested by other researchers, there is a clear need for more effectiveness studies from mobile integrated health programs in emergency medicine, to explore their development and the results they produce. The results presented here offer insight into the overall effectiveness of a large-scale program currently underway.

As populations continue to grow, municipal resources shrink, and hospital EDs continue to have limited capacity, the demand on traditional EMS will create significant problems. Alternative models, through mobile integrated health and community paramedicine, offer potential to improve EMS utilization while maintaining quality of care and better aligning patients with the appropriate level of care. Around the country, multiple demonstration projects are underway, but little evidence exists to support their impact on care delivery.

In this research, we found that the integration of a telehealth-based initiative with patient navigation to more appropriate care levels, creates significant reduction in ambulance-enabled ED utilization. Specifically, we found that the program resulted in a median 44-minute reduction in the unit back in service time (39 vs. 83 minutes). This equates to roughly 2.12 times greater productivity. We also observed a significant reduction in ED ambulance transports, from 74% to only 18%. These results come with little or no significant impact on clinical quality or patient satisfaction.

This study confirms that potentially unnecessary ambulance transports to the ED can be significantly reduced, which has significant financial and utilization impact on EMS agencies. We surmise that use of community paramedicine combined with telehealth and other mobile technology has potential to improve both EMS agency and overall emergency system capacity.

There are interesting financial consequences of this research. According to the Centers for Medicare and Medicaid, of the 107 funded “Health Care Innovation” awards, which recently ended their three-year funding term, only a few involve EMS.^19^ Based on our findings, we suggest that a significantly greater number of programs be implemented in rural and urban, large and small communities, to create meaningful change nationwide.

Implementing these programs will not be easy, and there are a number of barriers to alternative EMS models. Lack of reimbursement for non-ED transports is clearly significant. Medicare currently does not provide reimbursement unless the patient is transported to the ED.^20^ Although researchers have called for payment policy reform to include broader ranges of EMS transport options, they have not yet been adopted.^21^ In addition, the lack of reliable field triage criteria and paramedic assessment of medical necessity creates barriers.^22^–^27^ However, technological advancements such as telemedicine, real-time telemetry, and electronic health information exchange (HIE) have made it feasible for paramedics in the field and remotely located physicians to accurately assess, safely manage, and determine resource-efficient courses of action for patients.^28^–^29^ Reimbursement mechanisms for more proactive, alternative models of EMS deployment as well as telehealth will also need to be developed.

The evolution of mobile integrated health programs in EMS has developed rapidly. Within the last five years, dozens of programs have evolved to reduce ED utilization, unnecessary ambulance transports, and improve overall outcomes. The productivity gains we observed in this study should offer evidence to support further innovations in EMS as well as change in policy and reimbursement practices. We contribute to the literature by providing comparative effectiveness research from one of the largest EMS agencies in the country.

## CONCLUSION

A telehealth-enabled emergency medical services program reduced unnecessary ambulance transports by 56% to urban emergency departments, and put paramedic units back in service an average of 44 minutes faster.

## Figures and Tables

**Figure:**
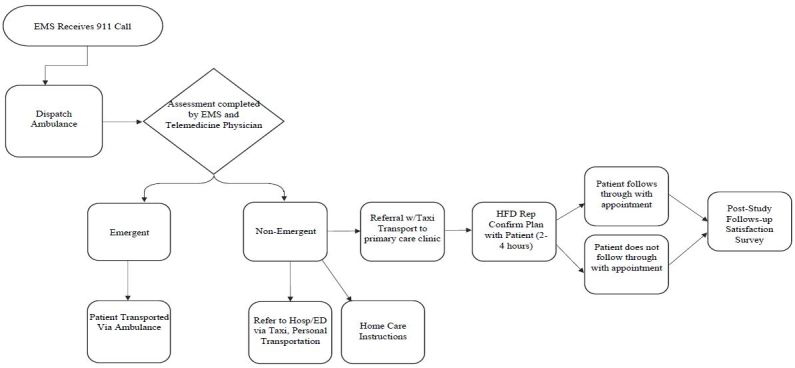
Study of intervention protocol flow chart. *EMS,* emergency medical services; *ED,* emergency department; *HFD,* Houston Fire Department

**Table 1:** Descriptive characteristics of intervention patients and control group in a study comparing the effectiveness of an alternative EMS telehealth delivery model relative to traditional EMS care.

Measure	Intervention	Control
Race/ethnicity		
White	17%	15%
Black/African American	58%	60%
Hispanic/Latino	17%	20%
All other	8%	5%
Matched measure		
Median age, IQR, y	44 (10)	45 (10)
Sex % female	55%	51%
Top 3 chief complaints		
% “Abdomen pain”	15%	17%
% “Sick”	25%	29%
% “Breathing”	20%	18%

*EMS,* emergency medical services, *IQR,* interquartile range

**Table 2:** Patient disposition intervention in an emergency telehealth and navigation program (ETHAN).

Patient disposition	N	% of total
Hospital ED with taxi	3,293	59%
Ambulance transport to ED	1,013	18%
Clinic referral with taxi	458	8%
Referral to PCP or home care	419	8%
Others (refusals, technical issues; no transport or referral)	387	7%
Total Sample	5,570	100%

*ED,* emergency department; *PCP,* primary care provider

**Table 3:** Outcome differences comparison in a pilot program that integrates mobile technologies and alternative patient navigation to improve EMS utilization and outcomes.

Outcome category	Measure	Control group	ETHAN (Intervention)	P
Ambulance utilization	Disposition to ED by ambulance (% ambulance transport)	74%	18%	<.001
Unit productivity	Total back in service time median minutes (IQR)	83 (20–140)	39 (27–90)	<.001
Quality of care	Mortality	0%	0%	na
Experience of care	Patient satisfaction	87%	88%	.250

*ETHAN,* Emergency Telehealth And Navigation Program; *ED,* emergency department; *IQR,* interquartile range
